# Different Neuroplasticity for Task Targets and Distractors

**DOI:** 10.1371/journal.pone.0015342

**Published:** 2011-01-31

**Authors:** Elsie Y. Spingath, Hyun Sug Kang, Thane Plummer, David T. Blake

**Affiliations:** 1 Brain and Behavior Discovery Institute, Medical College of Georgia, Augusta, Georgia, United States of America; 2 Graduate Program in Neuroscience, Medical College of Georgia, Augusta, Georgia, United States of America; 3 Department of Neurology, Medical College of Georgia, Augusta, Georgia, United States of America; Claremont Colleges, United States of America

## Abstract

Adult learning-induced sensory cortex plasticity results in enhanced action potential rates in neurons that have the most relevant information for the task, or those that respond strongly to one sensory stimulus but weakly to its comparison stimulus. Current theories suggest this plasticity is caused when target stimulus evoked activity is enhanced by reward signals from neuromodulatory nuclei. Prior work has found evidence suggestive of nonselective enhancement of neural responses, and suppression of responses to task distractors, but the differences in these effects between detection and discrimination have not been directly tested. Using cortical implants, we defined physiological responses in macaque somatosensory cortex during serial, matched, detection and discrimination tasks. Nonselective increases in neural responsiveness were observed during detection learning. Suppression of responses to task distractors was observed during discrimination learning, and this suppression was specific to cortical locations that sampled responses to the task distractor before learning. Changes in receptive field size were measured as the area of skin that had a significant response to a constant magnitude stimulus, and these areal changes paralleled changes in responsiveness. From before detection learning until after discrimination learning, the enduring changes were selective suppression of cortical locations responsive to task distractors, and nonselective enhancement of responsiveness at cortical locations selective for target and control skin sites. A comparison of observations in prior studies with the observed plasticity effects suggests that the non-selective response enhancement and selective suppression suffice to explain known plasticity phenomena in simple spatial tasks. This work suggests that differential responsiveness to task targets and distractors in primary sensory cortex for a simple spatial detection and discrimination task arise from nonselective increases in response over a broad cortical locus that includes the representation of the task target, and selective suppression of responses to the task distractor within this locus.

## Introduction

The adult brain learns to discriminate somatosensory, auditory, and visual forms through experience. This experience causes representational changes in primary sensory cortex [1]–[13]. To a first approximation, neurons that respond to stimuli associated with reward develop stronger responses throughout practice, whereas neurons associated with omission of reward exhibit weaker responses after experience. This learning rule implies that neurons carrying the most reward–relevant information, or those that respond strongly to the target and weakly to the distractor, will have their responsiveness enhanced [10], [12], [14]–[18], and that neurons are impacted by associational learning [11]. Additionally, sensory cortex activity has been demonstrated to comprise the signals upon which decisions are made [19]–[22], which, when combined with the aforementioned reward–association neuroplasticity rules, implies that a significant component of perceptual learning is dependent on sensory cortex changes.

To selectively reinforce the neurons that respond well to the target but not to the distractor, and not just those with strong target responses, several strategies may be used. First, the reinforcement process could be unimodal and selectively enhance the most informative neurons. This strategy, however, requires a reinforcement process that can differentiate between high levels of neural activity, or the neural response to the target, and lower levels of neural activity that are more informative about reinforcement, or neural responses that differentiate the target and distractor. It has been suggested that an underlying process is the pairing of neuromodulators with stimulus evoked activity. The neuromodulators, which are released in sensory cortex after stimuli associated with reward, act to potentiate the responses to task targets [23]–[29]. Although this mechanism is plausible and backed by data, it requires additional mechanisms to potentiate informative responses, and not just target stimulus responses.

The explanation of the neural mechanisms gets simpler if the reinforcement processes are bimodal. With one mechanism to enhance responses to stimuli associated with reward, or task targets, and a second mechanism to suppress responses to stimuli associated with omission of reward, or task distractors, the most informative neural signals will be enhanced. Although sensory discrimination experiments have observed suppression of responses to task distractors [10], [18], these experiments cannot distinguish between unimodal and bimodal reinforcement processes because the plasticity effects for the task target are confounded with those for the task distractor. Similarly, nonspecific plasticity effects that have been observed are not closely tied to reward, or omission of reward, associations because these effects have been observed in discrimination tasks.

To differentiate between these possibilities, we trained monkeys serially on detection and discrimination tasks. This training strategy isolates effects that occur when an animal associates a target stimulus with a reward from those that occur when an animal associates a distractor stimulus with omission of reward. The most–informative reinforcement rule hypothesis predicts that neurons that prefer task targets will strengthen on detection learning, and will continue to strengthen on discrimination learning. The bipolar reinforcement rule hypothesis additionally predicts suppression of responses to the task distractors upon discrimination learning. Our animals are implanted with custom cortical microarrays to allow daily population measurements to be collected so that representations may be tracked throughout the learning process [30], which enables serial learning experiments such as this one. The results demonstrate the necessity of this type of experiment, as our understanding of the reward association plasticity has to be reconsidered upon observing the results and incorporating them with existing work.

## Results

A series of behavioral tasks were designed to segregate different hypotheses about how responses of the most informative neurons are enhanced during sensory discrimination learning. An array of 64 microelectrodes was implanted into the primary somatosensory cortex of two adult Rhesus monkeys, and responses were allowed to stabilize for more than six weeks. Data, specifically receptive field maps and responses to calibrated skin indentations, were collected before each day's behavioral session. The same sensory stimuli that are presented during the behavior are tested prior to each day's behavioral session to minimize changes in attention and arousal that may occur throughout learning during the study.

**Figure 1:**
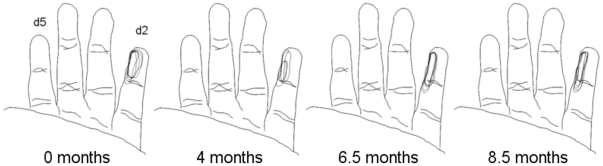
Consistency of mapped RFs over a period of 8.5 months indicate a stable implant. Each hand diagram shows five overlaid receptive fields from the same implanted electrode over a week.

The electrodes in our implants sample responses from the same skin surfaces throughout the study. An example that shows the reliability of this process throughout 8.5 months is shown in Figure 1. Receptive fields are mapped manually, but the person defining the receptive field is blinded to the identity of the implanted electrode to which she was listening.

Each electrode sampled multiunit data from the same cortical location throughout the study. 63 electrodes yielded somatosensory responses from the two animals. Of these, 18 were consistent and used in the four behavioral experiments in this study. From those 18 electrodes, 567 quantitative firing rate profiles were taken, and 542 receptive fields were measured.

**Figure 2:**
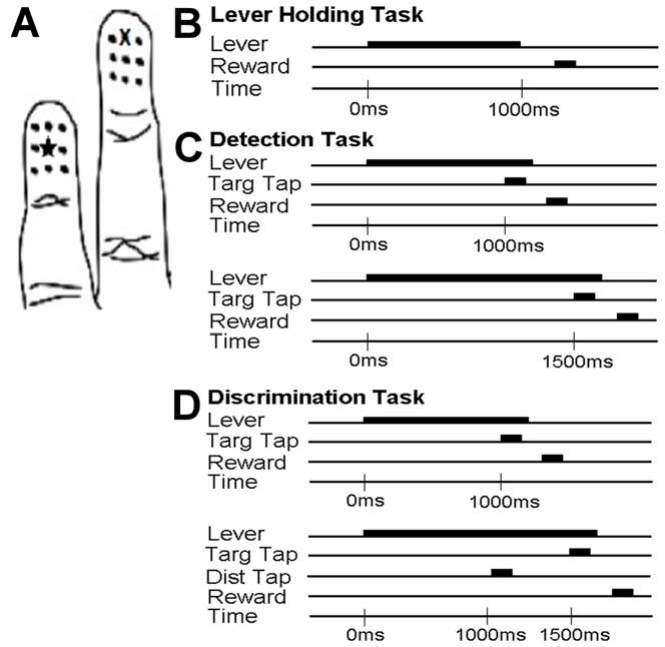
Hand marking and behaviors. A. Marked grid of sites on digits ensures spatial consistency when collecting data daily. For each experiment, one site is assigned to receive the target tap (x), and another site is assigned to receive the distractor tap (star). B. Lever holding task. The monkey must hold the lever pressed longer than the minimal hold time to get a reward upon lever release. C. Detection task. The monkey releases the lever in response to the 200 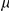
m target tap for a reward. The figure illustrates the two types of trials which are randomly interleaved. The target tap can occur at different times relative to the initiation of the lever hold. D. Discrimination task. The task requires releasing the lever after a target tap. As with the detection task, two types of trials are randomly interleaved. Distractor taps are presented on trials in which the target is presented at a later time. Except for the presence of the distractor tap on half the trials, the discrimination task is identical to the detection task.

Animals were pre–trained on a lever holding task. This task, shown in Figure 2B, required the monkey to press and hold a lever for a minimum hold time, after which a lever release triggered a reward.

After implantation and performance of the lever holding task for several weeks, animals were transitioned to the detection task. This task, shown in Figure 2C, requires animals to hold a lever press until a 200 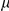
m tactile tap is delivered to the target skin location on the hand contralateral to the implant. The detection task was performed for two weeks after learning. The discrimination task is identical to the detection task, except that a distractor tap is delivered on trials in which the target is presented at 1500 msec, as shown in Figure 2D.

### Detection Task

**Figure 3:**
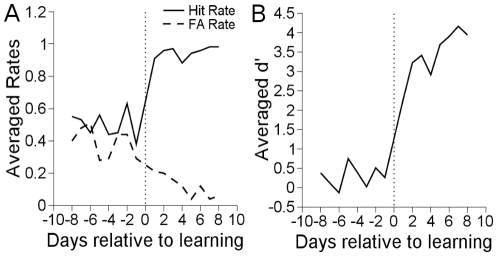
Behavioral performance in detection learning. A. Hit and false alarm rates for detection learning. Different experimental runs were aligned on the day of learning which typically featured a sharp break between hit and false alarm rates. B. d' averaged across experimental runs. d' increases as hit rate increases and false alarm rate decreases. In this and all subsequent daily learning graphs, data points do not occur on day zero. Day -1 is the pre-behavioral data collection session on the day of learning before learning occurred. Day 1 is the day after learning occurred. Day 1 and day -1 are temporally separated by one day.

The detection task was learned a total of four times in two animals. In all cases, significantly positive d' values indicated that the animals had learned the task. Animals typically performed the task for 1000 rewarded trials a day. The last day d' increased to greater than 0.6 (or p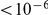) was considered the day the detection task was learned. d' is a signal detection metric that increases as the animal increases in hit rates or decreases in false positive rates [31]. Hit rates, false alarm rates, and d' are shown in Figure 3.

Firing rate responses to tap stimulation at skin sites were grouped into electrodes that sampled target site responses before detection learning, electrodes that sampled pre-distractor site responses before learning, and electrodes that sampled from neither the target or pre-distractor site, which were the control responses. During the detection task, one tactile motor tip statically indented each of the target site and the pre-distractor site by 500 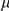
m. The motor tip on the pre-distractor site did not deliver any taps during the detection behavior, but did deliver taps once the discrimination behavior began. Recordings from neurons whose receptive fields did not include the target or distractor site were control responses.

To take advantage of the controlled sampling from using an implant, firing rate samples from each electrode prior to learning were paired with samples from the same electrode after learning. Eight samples were taken in the last eight days prior to learning, and these were compared with the first eight samples after learning. At the beginning of each experimental run, electrodes were grouped as target, distractor, or control, depending on whether the receptive field included a target site, distractor site, or neither in their receptive field. Target electrode responsiveness was evaluated based on the strength of the stimulus evoked response at that electrode to a tap at the target skin location. Distractor electrode responsiveness was determined analogously for the distractor tap. Control electrode responsiveness was evaluated based on the response to a tap of the same amplitude as the target and distractor tap delivered at a location in our sampling grid that maximized the tap response for that control electrode. Control skin sites were specific for each electrode, and constant for each behavioral series.

To determine overall target, distractor, or control responsiveness, firing rate data was averaged across the four experimental runs for each group of electrodes. Data before learning was compared to the data after learning with a two-tailed t-test. The target group contained samples from four electrodes that sampled target skin sites, the pre–distractor group contained samples from four electrodes that sampled pre–distractor skin sites, and the control group contained samples from eleven electrodes that were each sampled at their most sensitive location within our grid. This analysis consisted of 64 target, 64 distractor, and 176 control firing rate profiles.

**Figure 4:**
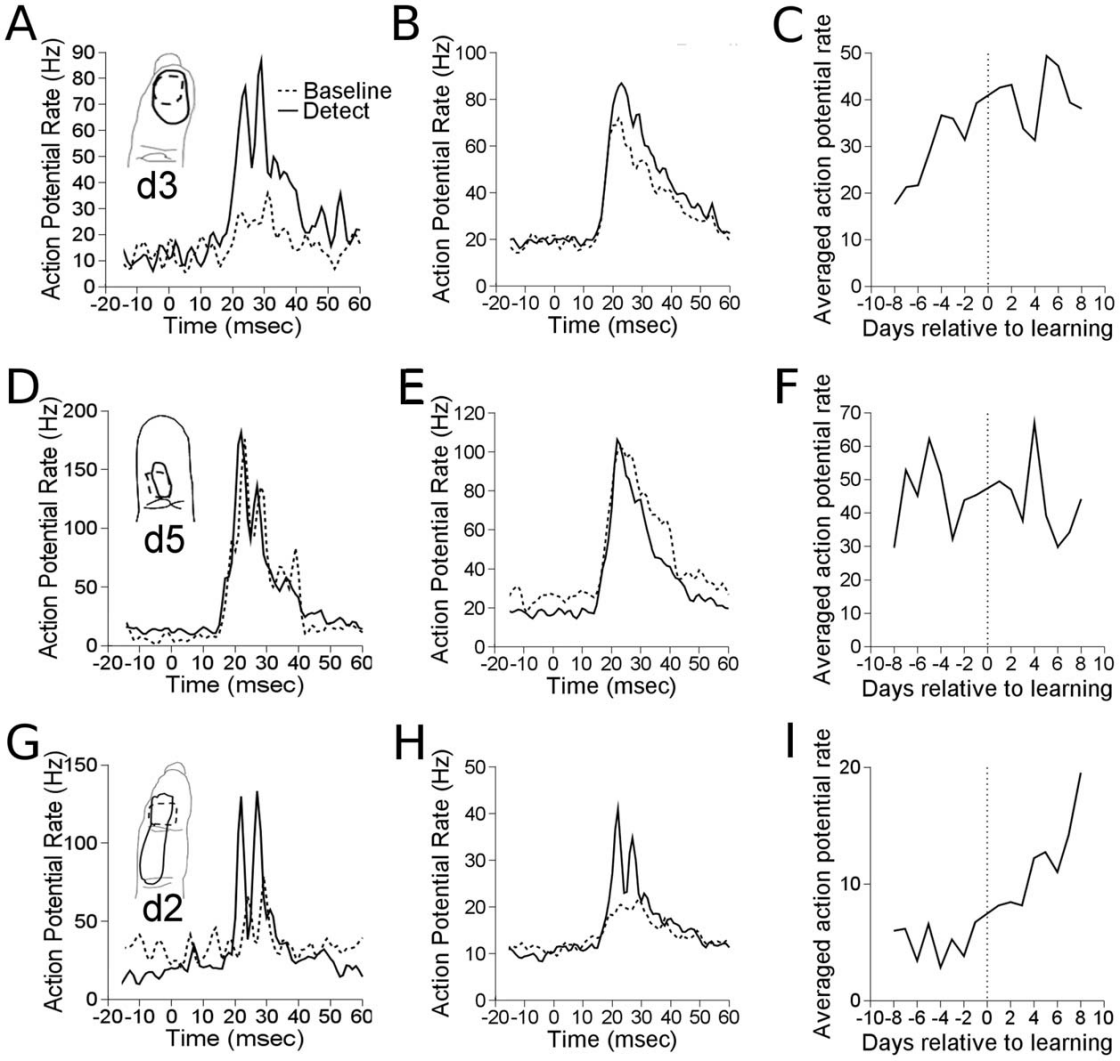
Effects of learning detection on firing rate. A. Single electrode example of target site. Peristimulus time histograms (PSTHs) are averaged over a week each before and after learning. Inlaid receptive fields show examples from before and after learning. B. Population average. All target site responses from all experimental runs are averaged by group. Dashed line shows data before learning, and solid line shows data after learning. C. Daily average across all experimental runs of the same measures in B. D. Single electrode example of pre–distractor site. E. Population average of pre-distractor sites. F. Daily averages of pre–distractor sites. G–I. As in A–C and D–F, for Control sites.

Upon learning detection, neural responsiveness to target sites increased from an average firing rate of 29.0 imp/sec to 40.6 imp/sec, and this was significant, as shown in Figure 4A–C (p
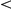
0.0065). Response strength to taps at the target site were, on average, 140% of the response strength before learning. Control electrodes also increased in responsiveness (p
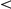
0.0005), as shown in Figure 4G–I. The responses to taps in the sensitive region of the receptive field for control electrodes after learning were 232% of the response strength before learning. Control responses increased from an average of 5.1 imp/sec to 11.8 imp/sec after learning detection. Neural responsiveness to pre-distractor sites did not exhibit a significant change in firing rate upon learning detection, as shown in Figure 4D–F. The control electrodes were divided into electrodes that developed responses to the target stimulus (n = 3), and those that did not (n = 8). The control responses to a stimulus within their original receptive field, and not the target skin site, were analyzed for plasticity. Both electrodes that developed target responses and those that did not significantly increased their responses after detection learning (p
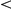
0.00003 and p
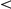
0.0011 respectively). Controls that developed target responses increased from 0.7 imp/sec to 6.6 imp/sec, and this was 927% of the responsiveness before learning detection. Those that did not develop target responses increased responsiveness from an average of 6.7 imp/sec to 13.6 imp/sec, 201% of the responsiveness before learning detection. The control electrodes all had clear tactile responses on all days, even though some did not yield clear responses to the 200 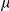
m taps on some days.

Receptive field areas were analyzed separately from the firing rate data. Receptive field data was grouped into two electrodes that sampled the target skin site only, two electrodes that sampled the pre-distractor site only, two electrodes that sampled both the target and pre-distractor site, and twelve electrodes that sampled control skin sites. In total, 32 target only, 32 distractor only, 32 target and distractor, and 192 control receptive field profiles comprised this analysis. More control electrodes were used for receptive field analysis than for action potential rate analysis because some electrodes had receptive fields that were accessible using manual mapping methods but not located within our grid used for that experiment. These were typically not close to the plane of the ventral surface of the hand. The same days were compared in the receptive field analysis that were compared in the firing rate analysis.

**Figure 5:**
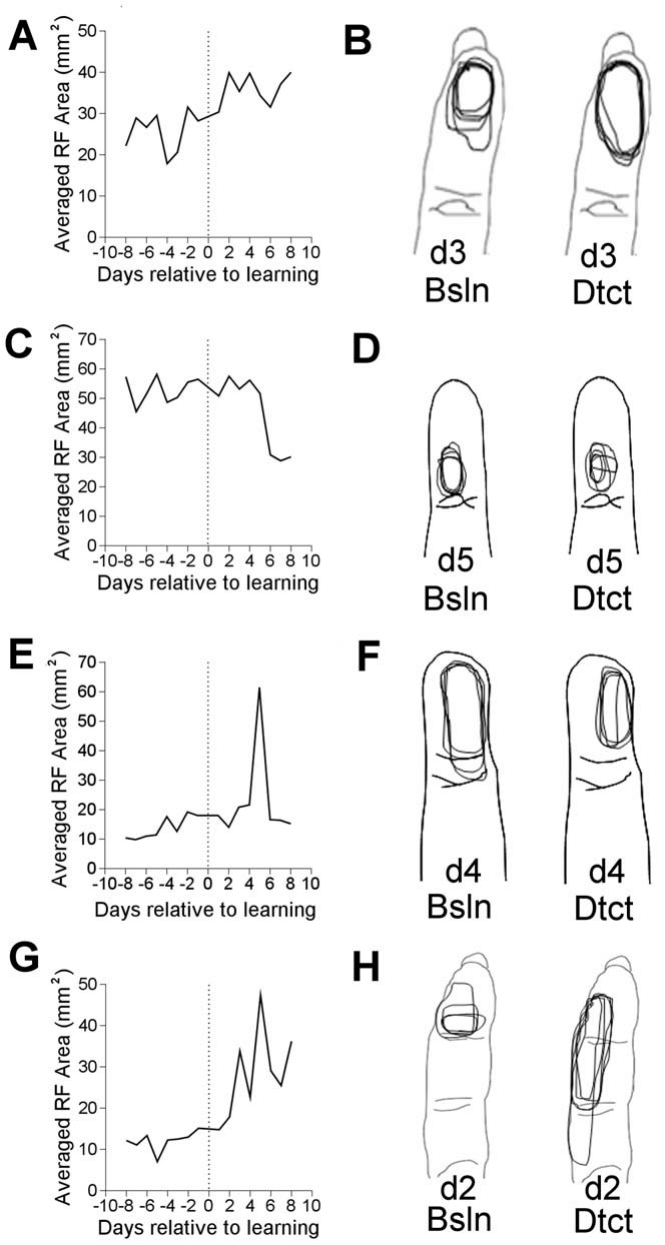
Effects of learning detection on receptive field area. A. The average area of all receptive fields that contain only the target skin site. B. A single electrode example of groups of receptive fields before and after learning. This electrode sampled a receptive field containing only a target. C–D. As in A–B, but electrodes sampled receptive fields containing only the pre–distractor site. E–F. As in A–B, but electrodes sampled both the target and distractor. G–H. Control data. Each digit has overlaid 5 days of mapped receptive fields.

The changes in receptive field area upon learning detection were similar to the firing rate changes, and are shown in Figure 5. Target only receptive fields exhibited a significant increase in receptive field area upon detection learning (p
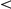
0.0004). These receptive field areas increased from an average area of 25.6 mm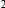 to 36.0 mm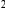, and the areas after learning were 140% of the before learning areas. Neither receptive fields that contained both the target and pre-distractor sites, nor receptive fields that only contained the pre-distractor sites exhibited significant changes. All receptive fields that included targets, which is the sum of the cortical locations with receptive fields that included only the target and those that included the target and distractor, increased from an average of 21.0 mm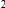 to 29.4 mm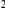. The areas after learning were 140% of the areas before learning (p
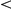
0.02). Control receptive fields exhibited a significant increase in receptive field area. They increased from an average of 11.2 mm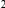 to 27.7 mm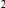, and were 247% of the areas before learning (p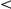 0.001).

### Discrimination Task

**Figure 6:**
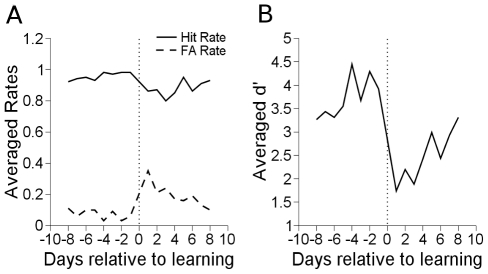
Behavioral performance in discrimination learning. A. Hit and false alarm rates for discrimination learning. Different experimental runs were aligned on the day the distractor was introduced. B. d' averaged across experimental runs. d' increases as hit rate increases and false alarm rate decreases.

After two weeks of performing the detection task, the distractor tap was initiated in experimental runs. Learning consisted of suppressing behavioral responses to the new task distractor, shown in Figure 6. Learning always occurred on the first day, and is noted by the changes in false alarm rate on subsequent days.

**Figure 7:**
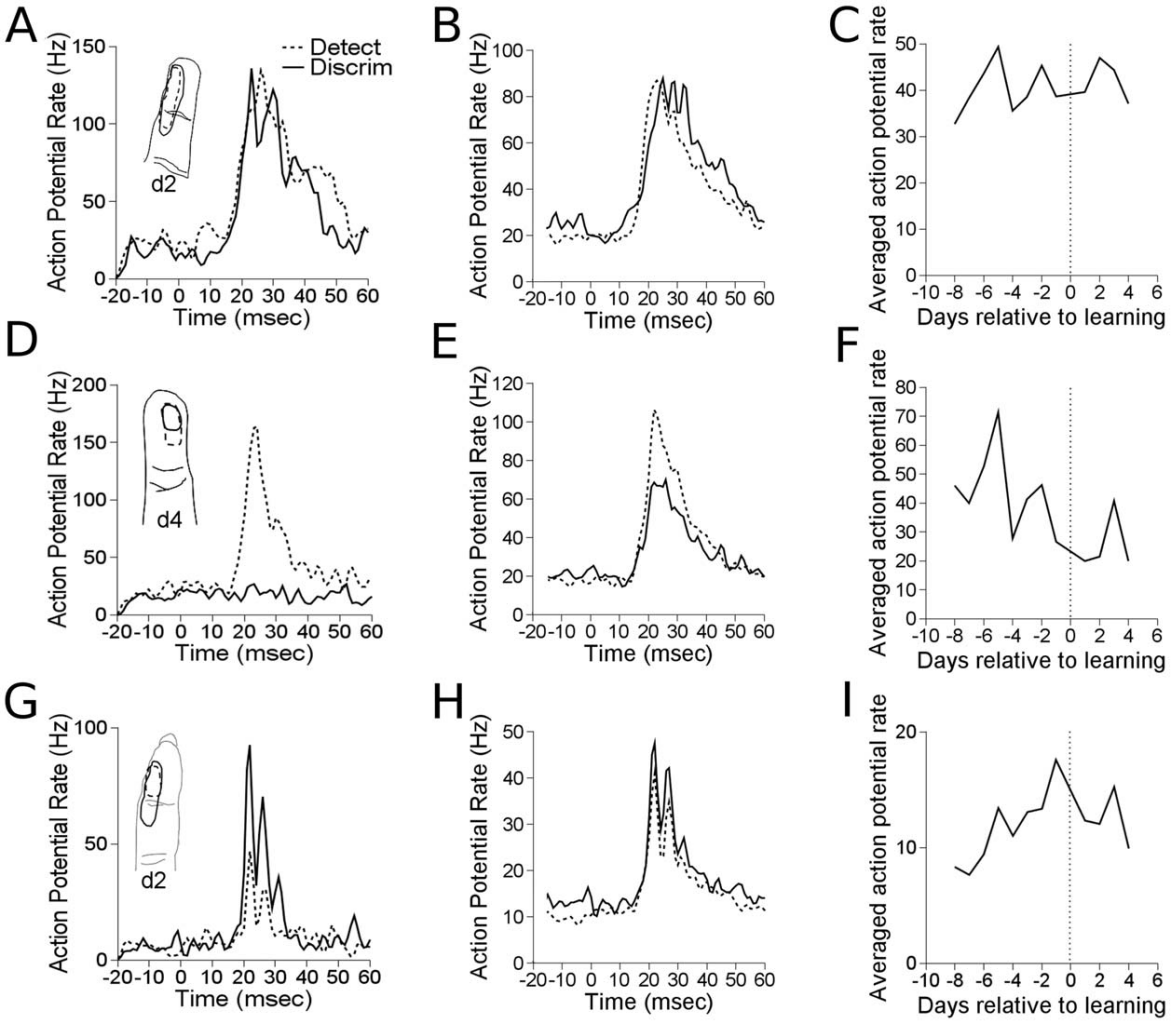
Effects of learning discrimination on firing rate. A. Single channel example of target site. Peristimulus time histograms (PSTHs) are averaged over a week each before and after learning. Inlaid receptive fields show examples from before and after learning. B. Population average. All target site responses from all experimental runs are averaged by group. Dashed line shows data before learning, and solid line shows data after learning. C. Daily average across all experimental runs of the same measures in B. D. Single channel example of pre–distractor site. E. Population average of distractor sites. F. Daily averages of pre–distractor sites. G–I. As in A–C and D–F, for Control sites.

In comparing detection with discrimination, the only significant changes in tap responses that occurred were found in distractor responses. Neural responsiveness to distractor sites was significantly suppressed (two-tailed t-test, p
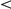
0.05, Fig. 7D–F). The average firing rate response to distractor sites decreased from 44.0 imp/sec to 25.5 imp/sec after learning discrimination. The response strength was only 58% of the response prior to learning. Target and control responses did not significantly change in the first week after learning discrimination. Comparison of neural responsiveness to specific skin sites in detection and discrimination conditions took into account all relevant electrodes across the four experimental runs that continued into discrimination. Eight days' of data before discrimination learning were compared with four days after discrimination learning. Only four days after discrimination learning were compared because one of the experimental runs had to be stopped early in the first animal. In other experimental runs, at least eight days of discrimination data were collected. Firing rate data from each electrode across the twelve comparison recordings were averaged, and then data was pooled across electrodes by condition. Four electrodes were used for target comparisons, four were used for distractor comparisons, and eleven were used for control comparisons. In total, 48 target, 48 distractor, and 132 control firing rate profiles comprised this analysis.

**Figure 8:**
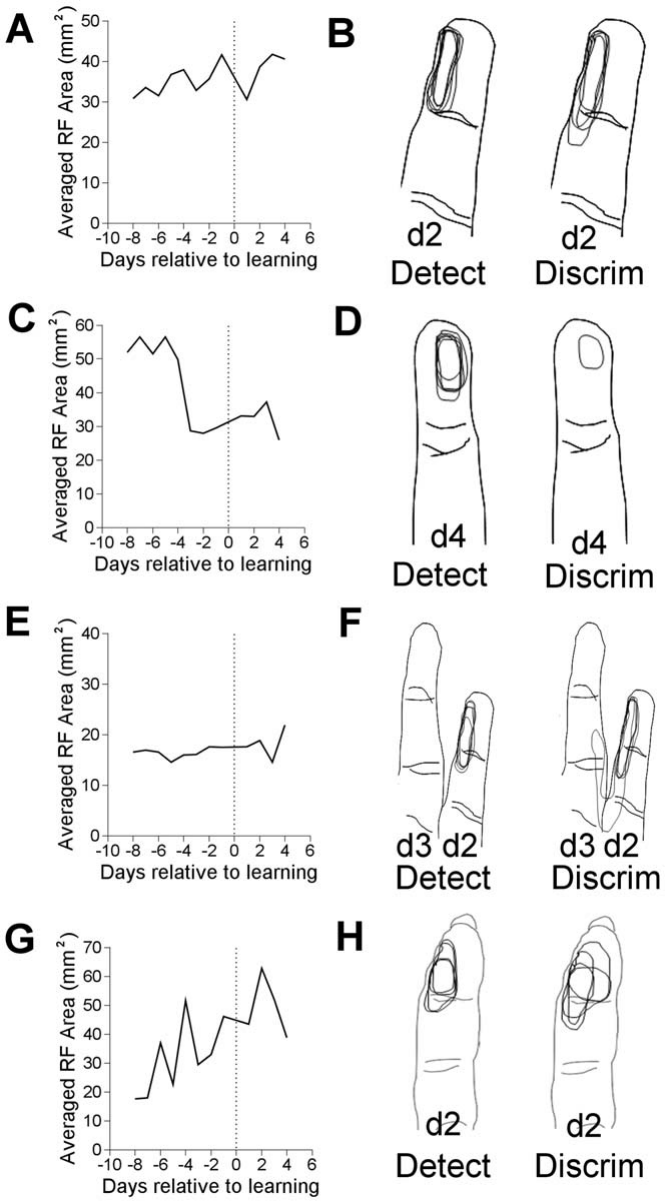
Effects of learning discrimination on receptive field area. A. The average area of all receptive fields that contain only the target skin site. B. A single electrode example of groups of receptive fields before and after learning. This electrode sampled a receptive field containing only a target. C–D. As in A-B, but electrodes sampled receptive fields containing only the pre–distractor site. E–F. As in A–B, but electrodes sampled both the target and distractor. G–H. Control data. Each digit has overlaid 5 days of mapped receptive fields.

Learning discrimination caused an increase in the control receptive field areas. They increased from an average area of 31.9 mm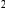 to 49.2 mm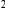 and were 154% of the areas before learning discrimination (p
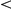
0.04). All other group changes were nonsignificant, and are shown in Figure 8. Data across all four experimental runs were grouped into two electrodes that sampled the target site only, two that sampled the distractor site only, two that sampled the target and distractor site, and twelve that sampled control sites. Data was averaged and compared with a two-tailed t-test. Eight days' of data before learning discrimination were again compared with four days' of data just after learning discrimination. A total of 24 target only, 24 distractor only, 24 target and distractor, and 144 control receptive field profiles comprised this analysis.

### Effects from Baseline to Discrimination

Overall changes from the beginning of an experimental run through to the very end were assessed. Eight days' of data from the baseline, pre-detection, condition were compared with four days' of data at the end of the discrimination period, and statistical significance assessed with a two-tailed t-test. As with other data, statistical significance was assessed after all action potential firing rates and receptive field areas were averaged and take into account all relevant electrodes across the four experimental runs that went from baseline to discrimination. Data from four electrodes constituted the target group, data from four electrodes constituted the distractor group, and data from eleven electrodes constituted the control group. In total, 48 target, 48 distractor, and 132 control firing rate profiles comprised this analysis.

**Figure 9:**
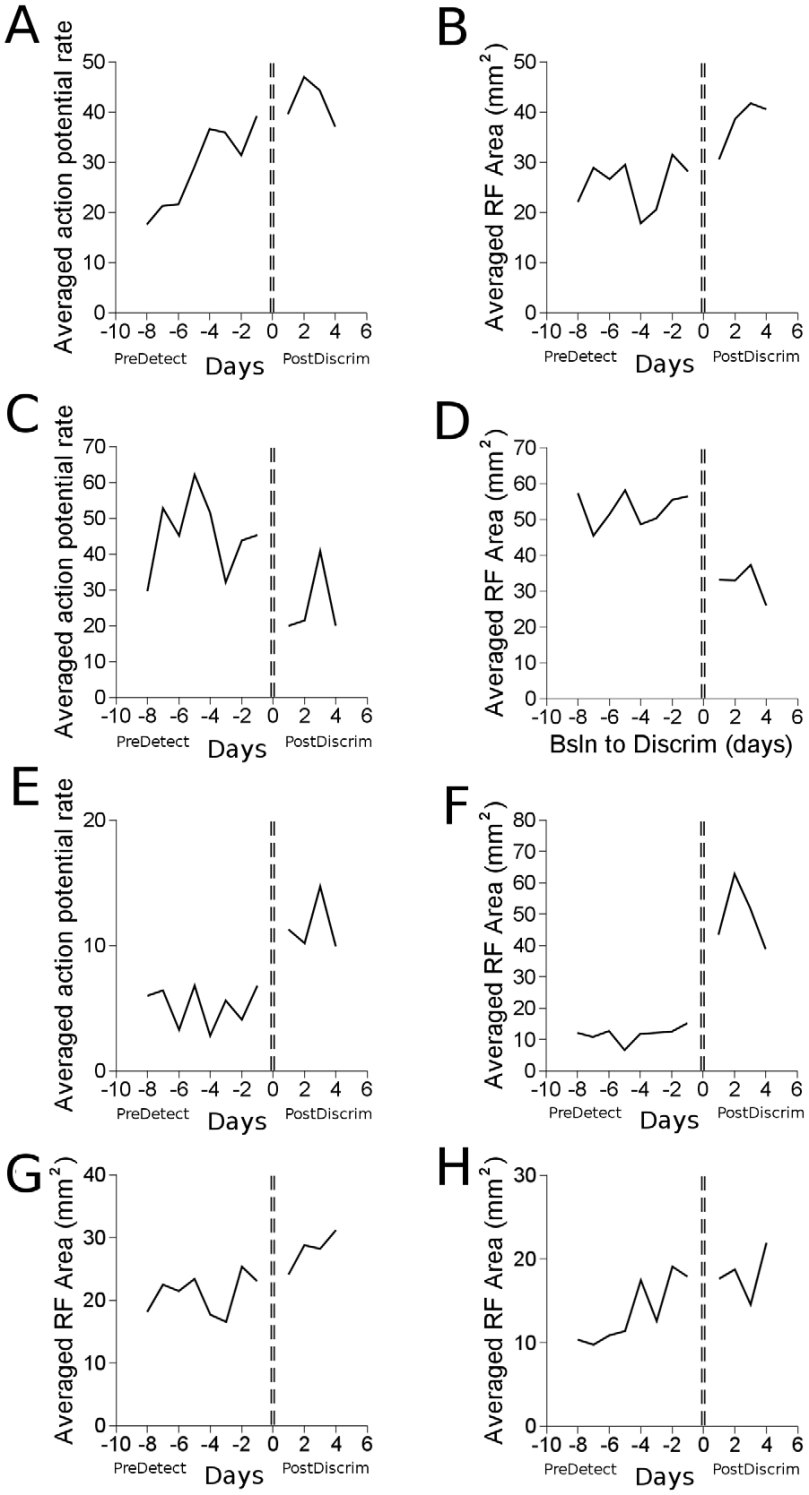
Firing rate and receptive field size effects from the beginning of the experimental runs (PreDetect) to the end (PostDiscrim). A. Averaged target firing rate response to tap at target skin site. B. Averaged receptive field area from electrodes that sampled only the target. C. Averaged distractor firing rate response to tap at distractor skin site. D. Averaged receptive field from electrodes that sampled only the distractor skin site. E. Averaged control tap response. F. Averaged control receptive field area. G Averaged receptive field area over all electrodes that sampled targets (average of B. and H). H. Averaged receptive field area over all electrodes that sampled both target and distractor.

At target sites, Figure 9A, neural responses increased from an average of 29.0 imp/sec before detection learning to 42.0 imp/sec after discrimination. The response after discrimination was 145% of the rates before detection learning (p
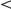
0.015). At distractor sites, shown in Figure 9C, average rates decreased from 45.2 imp/sec to 25.5 imp/sec, and the responses were 56% of rates before detection learning (p
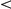
0.015). At control sites, shown in Figure 9E, tap responses increased from 5.1 imp/sec to 12.4 imp/sec and were 243% of before detection learning levels (p
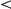
0.00005).

When comparing receptive field areas, two electrodes sampled the target skin site, two sampled only the distractor skin site, two sampled both the target and distractor skin site, and twelve sampled control sites. The same days were compared in the receptive field analysis as were compared in the firing rate analysis. A total of 24 target only, 24 distractor only, 24 target and distractor, and 144 control receptive field profiles were compared in this analysis.

Receptive fields that included only the target site increased from an average area of 25.6 mm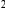 to 37.8 mm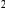, and were 148% of their area before detection learning (p
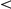
0.003). Receptive fields that included both the target and distractor skin site did not change significantly. Receptive fields that included only the distractor decreased from 52.8 mm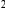 to 32.3 mm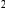. The final receptive field areas after discrimination were 61% of their before detection learning area (p
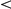
0.00003). If all receptive fields including the target are analyzed, which is a combination of two groups already presented, area increased 134% from 21.0 mm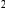 to 28.0 mm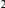 (p
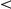
0.005). Control receptive fields increased to 438% of their area before detection learning, from 11.2 mm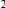 to 49.2 mm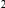 (p
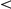
0.000002).

## Discussion

This work supports the hypothesis that reinforcement processes in sensory discrimination learning is bimodal, having one mode for plasticity that results when a task target is associated with reward, and a second mode when a task distractor is associated with omission of reward. The hypothesis that reinforcement processes are unimodal may be rejected. This work also introduces a new species to the implant studies of adult learning induced sensory cortex plasticity. Prior studies had used owl monkeys [10], [12], [18]. The most surprising finding is that by the end of study, control locations are potentiated more than locations containing responses to the task targets. The nonselective response enhancement has been observed in multiple prior studies [10], [12], [18], but never isolated to the effects associated with reward. The hypothesis that these nonselective increases in response strength are temporary and are caused by changes in the animal's state of attention or arousal may be rejected because the effects are not seen at the cortical locations with neural responses to the task distractors. We offer multiple alternative explanations, which are not mutually exclusive. The first is that the neuroplasticity is guided by feedback from hierarchically higher sensory cortices, like areas 1 and SII. A simple possibility is that the target neural activity is projected to these higher areas, which in turn feedback to the lower areas to direct the plasticity. These feedback projections may also be involved in the preparatory set of the animal [32]. The preparatory set refers to the set of neural activity involved in readiness and anticipation of experience. The preparatory set would be expected to include biases in activity from higher somatosensory cortices [33]. The involvement of the preparatory set in cortical plasticity may explain the topographic, task specific transfer of learning seen in somatosensory studies [34], and the activation of primary sensory cortex in fMRI studies during mental imagery [35] or during expectation of a task relevant stimulus [36]. Another possibility is the spread of plasticity from extrasynaptic neuromodulator release of noradrenaline [37]. Noradrenergic terminals project into the extrasynaptic space more often than in the synaptic space, and their spread to nearby cortical locations could be involved in the nonselective plasticity effects observed in the detection and discrimination task. Noradrenergic releasing neurons are known to have neural activity related to task performance [38]. Further work will be required to sort out these possibilities.

Irrespective of the neural signal causing the nonselective increase in responsiveness, the differential change between control and target sites requires consideration. Changes in responsiveness from before detection learning until after discrimination learning were stronger at control locations than at target locations. One possibility is that neural activity partially blocks some aspect of the nonselective increase. Similar effects have been observed in rodent auditory cortex, in which early plasticity is robust at cortical locations that did not respond to the task target *a priori*, but suppression is noted at cortical locations that did respond to the task target [39]. A second possibility is that selection bias causes this result. Locations that responded to the task target and distractor were chosen because they had clear responses prior to study, and the leftover sites were control sites. As a population, the control sites were less responsive before study, and this selection bias could contribute to the differential plasticity observed comparing these locations and the target responsive locations. Further work should sort out whether the neural activity in response to the task target blocks the otherwise nonselective enhancement of responses or not.

A theoretical advantage of the non–selectivity in reward–association neuroplasticity relates to sensory system null spaces. Learning induced plasticity, which contains mechanisms to increase and decrease responsiveness, could marginalize representations of the sensory epithelia that are not typically used in reinforcing behaviors. For example, the hand map in the primate is incomplete in its representation of hairy skin [40]. Without some non–activity–dependent mechanism to potentiate very weak responses, these marginalized representations could not be potentiated. The brain elevates the activity of all potentially relevant cortical locations in response to an association with reward.

The selectivity in plasticity associated with the task distractor is easier to fit with existing data. Robust stimuli during a highly attended task that are not associated with reward are selectively suppressed perceptually [41] and physiologically [10]. In some cases, response suppression dominates the population response [8]. Further examples of this come from our prior work [10]. In tasks in which owl monkeys learned auditory frequency discrimination tasks, significant suppression of responses to all stimuli occurred prior to task learning. This suppression was plausibly caused by neural activity during task performance that the animal did not associate with reward because the task was not yet learned. Upon task learning, non–selective enhancement of responses occurred, followed by suppression of responses that was stronger for distractors than for task targets. The data in our study suggest that the plasticity that leads to suppression of task distractor responses is dependent on stimulus–evoked activity, and is stronger for stimuli not associated with reward. The cellular mechanisms associated with this suppression are not defined, but may relate to the specific set of neuromodulators that are present when the activity is evoked. Work in rodent barrel cortex has suggested LTD at cortico-cortical synapses plays a role in suppression of cortical responses [42], and its selectivity and activity dependence fit well with our observations.

A concern in any such study is the possible effect of electrode movement. It is clear that the exact neurons sampled changes over study, because the same single units are not present from start to end of study. Our approach is to sample from the same cortical position with the understanding that movement of the neuropil relative to the electrode can change sampling. Movement of the electrodes may be expected to add variance to our measures, but for it to impact our outcomes it would have to be a source of bias. Electrode movement would have to differentially impact the electrodes that sample from targets, distractors, and control sites as the changes are in opposite directions at appropriate times in detection and discrimination learning, and electrode movement would have to exert this bias consistently across four different learning progressions in two animals. For these reasons we feel it unlikely that the results of this study are in doubt because of potential electrode movement. Further, we have been unable to find any systematic shifts in receptive fields of neurons that would be expected from continual changes in electrode depth in area 3b, a cortical area that lies largely orthogonal to the brain surface so that depth changes in electrode position would move the electrode across columns.

An apparent discrepancy exists in how these observations dovetail with those in other studies, and particularly in single unit studies in macaque visual cortices [43], [44] which suggest that plasticity is limited to higher cortical areas, and demonstrate that plasticity in lower visual cortical areas have minimal contributions to perceptual learning in their tasks. Our nonhuman primate studies, in contrast, find plasticity relating to association with reward and omission of reward in primary auditory and somatosensory cortex [10], [12], [18]. This apparent discrepancy disappears on consideration of the evidence presented here. If a cortical area contains neurons that differentiate task targets from distractors, then our work would predict that learning–induced plasticity would be observable in that area. If a cortical area contains neurons that are sometimes associated with reward, and other times associated with omission of reward, then robust learning–induced plasticity would not be observable in that area. In any case, nonselective response enhancement shortly after learning is a powerful effect, and should be observable even in tasks in which neural populations associated with reward and omission of reward are not cleanly delineated in that cortical area [43].

Our data also documented parallel changes in receptive field size and responses to a tap delivered in the central portion of the receptive field. Our quantification of receptive fields was performed manually using a constant threshold for determining the boundaries of each receptive field. Our results are consistent with a model in which the responses at a cortical location are scaled up or down, and are not necessarily indicative of receptive fields changing the relative contributions of their inputs. These effects suggest neuroplastic changes in response to a single stimulus, the task target or distractor, have effects on responses to all stimuli within the local cortical area sampled for the determination of the receptive field. The non–selective increases in receptive field area that occur during detection learning must increase the distance across which overlapping receptive fields can be found. The re–shaping of overlap in responses in the cortex may have functional implications for disorders such as focal hand dystonia [45].

In conclusion, our work extends prior work on neuroplasticity in sensory discrimination learning. The reward–association of the task target results in non–selective increases in response strength and receptive field size. Existing target responses mildly strengthen, and some new cortical locations begin to respond to the task targets. In discrimination learning, the association with reward–omission of the task distractor results in suppression of response strength and decreases in receptive field size only at cortical locations that represent the distractor. As a result of these effects, selectivity in sensory discrimination plasticity is principally a function of the distractor stimuli used to contrast with the target.

## Methods

Ethics statement: Animal welfare was regulated by the Institutional Animal Care and Use Committee at the Medical College of Georgia under animal use protocol numbers 05-12-753 and 08-11-128. This study was carried out in strict accordance with the recommendations in the Guide for the Care and Use of Laboratory Animals of the National Institutes of Health. Within the considerations of procedures necessary to achieve scientific goals, animal suffering was minimized. Behavioral training was accomplished via food reinforcement without altering the weekly average of the daily intake of food. Surgeries were performed using aseptic technique in approved surgical suites, and anaesthesia and analgesia was carried out under the direct supervision of the clinical veterinarian. Animals were provided with environmental enrichment designed by the clinical veterinarian.

### Physiological Recordings

All data in this work was obtained from two adult, male Rhesus macaques weighing 4–7 kg. They were each implanted with an array of 64 microelectrodes. The microelectrodes were implanted into the somatosensory cortex. The somatosensory cortex was localized physiologically with microelectrode penetrations in surgery under barbiturate anesthesia to localize cutaneous somatosensory digit responses in the central sulcus, with the search for responses initiated at +6 mm anterior, and 24 mm lateral. Electrodes were implanted into area 3b and area 1 in the first animal, and into area 3b in the second animal. Data from both areas are pooled for this study. In the first animal, although electrodes were implanted at depths in the central sulcus consistent with areas 3b, dimpling at the implantation site pushed area 1 down towards area 3b so that the two areas were not well separated using Nissl stains. In the second animal, the areas were cleanly separable using Nissl stains, and recordings were all in area 3b. Microelectrodes were parylene–insulated iridium or parylene–insulated platinum–iridium electrodes that tapered from a 40 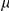
m diameter to an exposed electrode tip that ranged from 5–7 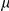
m long. This length of tip exposure was used to allow sampling from the smaller cell bodies present in sensory cortex [46]. Electrode depths were optimized for recording in the six week period after implantation surgery. After that point in time, electrodes were left unmoved for the remainder of the data presented in this work. Cortical implants are adapted from methods described previously [30]. Significant alterations to this method consisted of adding a fluid drain to relieve potential hydrocephalus (M. Tanifuji and N. Miyakawa, personal communication), removal of the dura in surgery in the areas of electrode penetration, and the replacement of cyanoacrylic bone cements with INFUSE bone graft (Medtronic, Minneapolis, MN).

Thresholds were set manually on each channel for multiunit data. Thus far, we have not been able to sustain adequate populations of single units for plasticity studies that require daily studies of the same populations. Multiunit thresholds were set so that spontaneous rates were roughly 10–20 Hz, which generally meant thresholds were close to 3.75 standard deviations. Channels, or electrodes, were not included for recording unless they had clear receptive fields in manual mapping, and each electrode that was used for analysis was checked every recording day, in most cases for many months. Sites were not sorted into slowly adapting type I, rapidly adapting, and Pacinian based on the limited stimulus set collected. The experimental focus was on changes in response to a constant stimulus set over a range of skin locations.

Somatosensory stimuli were delivered via custom–built tactile motors under LVDT displacement–feedback control. Each tap delivered from the motors was a single period of a 40 Hz raised sinusoid with a phase of -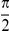 at its start and lasting 25 msec. More simply, a smooth tap with zero first derivative at the start, end, and midpoint. Neural responses to motorized taps were recorded before the day's behavior, during the behavior, and after the behavior in awake Rhesus macaques. This work only presents data collected before each day's behavior. Therefore, this data is largely free of contamination from alterations in arousal, motivation, and attention that obviously change during behavioral sessions each time an animal learns a new sensory discrimination that leads to changes in reinforcement.

Receptive fields were also defined using handheld 1 mm rounded glass tipped probes. Skin areas were included in cutaneous receptive fields if just–visible indentations of the skin evoked consistent audible responses in 250–10,000 Hz filtered voltage signals from the electrode. Calibration of this method with displacement controlled stimuli has determined that this threshold is under 100 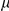
m. Stronger stimuli were used to map deeper or weaker contributions to the receptive fields which were separately noted. Pacinian input was determined by poorly localized, highly sensitive inputs to the glabrous skin, and hairy skin inputs were determined by responses to movements of isolated hairs. Trapezoidal skin indentations were not used to separate rapidaly adapting (RA) and slowly adapting (SA1) inputs, and recent evidence casts doubt on separate processing channels for SA1 and RA inputs in primary somatosensory cortex [47]. If the cortical locations responded to skin sites in our study grid, they were used. Using the Reconstruct software (Synapse Web, Austin, TX), receptive field boundaries were drawn over images of the hand and digits, and receptive field sizes were calculated by the software. Collection of automated receptive fields is not trivial in the somatosensory system, although it has been performed over a limited glabrous skin surface for peripheral afferents [48], and over planar surfaces in central neurons [49]. Receptive field maps over highly curved portions of the finger may be derived easily manually, but are especially challenging to do in an automated setup.

The person mapping receptive fields was the same throughout all studies. This person was blinded to the identity of the electrode being mapped, and the electrodes were always mapped in random order to prevent bias when mapping receptive fields.

### Stimulus Presentation

Tap stimuli were presented to the animal's digits in two basic contexts: to collect spiking data quantitatively outside of the rewarded behavioral context, and to present behaviorally relevant stimuli during the behavior. During presentation of any tactile stimuli and throughout the behaviors, the animal's hand and fingers were immobilized with a cast mold to ensure that the stimuli were presented and received in a consistent manner. The motorized tip was always lowered until barely touching the skin and then indented 500 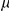
m into the skin before delivery of any taps.

Outside of the behavioral context, 50 taps were each presented at tap displacements of 100 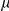
m, 200 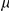
m, and 400 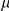
m. All data presented in this study were collected in this manner, before each day's behavior began. During collection of this data, the animal was seated passively and was not performing any behavioral tasks. Only data from the 200 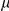
m taps were used in this paper, as these taps were physically identical to the taps that were used in the operant behavior. Each tap had the shape of one period of a 40 Hz sinusoid, with zero first derivative at its start, end, and midpoint.

During the behavior, the target or distractor tap that was presented was a single 200 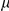
m tap. Displacements were continuously monitored via the LVDT sensor displayed on an oscilloscope. Human discriminative thresholds for longer 40 Hz stimulation is under 20 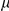
m, and the stimuli were perceived as clearly discernable.

### Animal Behavior

Prior to beginning experimental behavioral tasks, animals were pre–trained to perform a lever holding task constructed to mimic the operant component of the detection and discrimination tasks. This timed task consisted of holding a lever down for at least 1000 ms, releasing the lever, and then receiving a food reward triggered by the lever release. A 1000 ms intertrial interval prevented the animal from beginning the next trial immediately after completion of the previous trial. During the lever holding behavior, no taps were presented to the animal's digits in conjunction with any part of the behavior. However, before the lever holding task began for the day, data was collected daily by presenting a series of taps to each site on a grid of sites (Fig. 2). The grid was marked on the digits in permanent ink, and refreshed periodically. Neural responses to tap presentation were collected while the animal was passively seated. Data collected during the lever holding period was used for the baseline condition, and at least 10 days' worth of data was collected before proceeding to begin the detection task.

The detection task consisted of presenting a target tap at a pre–determined target site to which the animal could respond by releasing the lever for a food reward. The target tap was randomly presented at one of two time points, 1000 ms or 1500 ms. As the animal was pre–trained to release the lever around 1000 ms, presentation of the target tap around this time aided learning of the new task. The randomized timing of the target tap presentation prevented the animal from being able to perform the task solely by timing the behavioral response. The animal then learned to detect the target tap by trial and error. Throughout this task, a second motor tip was present at a 500 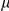
m indentation at the future distractor site. This second motor never presented any tap stimuli during the detection task.

The responses to the target on trials in which the target was presented at the earlier time were categorized as hits, misses, false alarms, or early errors. A hit is a correct response after a target stimulus was presented at 1000 ms and occurred when the animal released the lever within 500 ms after presentation of the target tap. A false alarm occurred when the animal released the lever between 1000 and 1500 ms, and the target for that trial would have occurred at 1500 ms. A miss occurred when the animal released the lever between 1500 and 2000 ms, and the target had been presented at 1000 ms. Misses and false alarms were always followed by a brief timeout. Early errors occurred when the animal released the lever before 1000 ms, that is, before a tap was ever presented. Early errors were discarded for behavioral analysis. The trials in which the target was presented at the later time were used only to classify false alarms, because once the target was not presented at the earlier time, the rest of the trial was not random.

The discrimination task only differed from the detection task in that an additional distractor tap was presented before presentation of the target tap. The two trial types, randomly interleaved, were a target tap presentation at 1000 ms, or a distractor tap presentation at 1000 ms followed by the target tap at 1500 ms. The animal only needed to continue responding to the target tap and ignore any distractor taps to receive a reward. Distinguishing the target tap from the distractor tap was learned by trial and error. The hits, misses and early errors are the same as in the detection task. A false alarm is defined as when the animal released the lever after presentation of the distractor tap at 1000 ms. Misses and false alarms were always followed by a brief 800 ms time–out, during which the animal would be unable to initiate another trial. Hits on trials on which a distractor tap was delivered were not counted as hits for the calculation of hit rate, because once the target is not delivered at the earlier time, the rest of the trial is not deterministic. Second window hits were used in the calculation of false alarm rate.

An experimental run consisted of a series of three behaviors: lever holding, detection, and discrimination. Target and distractor sites were determined at the beginning of each experimental run and remained the same until the end of the run. When beginning a new experimental run, a different set of target and distractor sites were independently chosen. Each target and distractor tap caused a tap response in one implanted electrode prior to the behavioral series. Control sites were in the center of the receptive field of an implanted electrode that did not respond to either the target or distractor tap prior to the behavioral series. Target and distractor sites were not always on the same digit and were not always in close proximity to each other. These skin sites could be on any digit, regardless of whether a digit had a target or distractor site on it in previous runs or in the run to be executed.

d' was calculated using hit rate for trials in which the target tap was presented at 1000 msec, and the false alarm rate in which the target was presented at 1500 msec. d' is calculable as the difference in the cumulative normal distribution corresponding to these two probabilities, which was calculated with the *norminv* function in Matlab (Mathworks, Natick, MA).

### Data Analysis

Neural response to a tap stimulus was calculated by subtracting baseline activity from evoked activity. Baseline activity was defined as the activity present from 20 ms before delivery of a tap to 10 ms after tap delivery, and evoked activity was defined as the response present from 15 ms to 45 ms after tap delivery. The conduction delay times from the peripheral nerve prevent earlier latencies, and most of the action potential energy occurs prior to 45 ms after the stimulus onset. To calculate whether firing rate changes were significant between baseline, detect, and discrimination conditions, a population of responses before and after learning were defined, and compared using a two-tailed t-test. The population consisted of as many days as were available from all experimental runs. Recordings over all the experimental runs were averaged. Then, the averaged before and after recording days constituted the samples to be compared in the t-tests. Receptive field area data was similarly analyzed.
